# The Role of Epigenetic Factors in the Pathogenesis of Psoriasis

**DOI:** 10.3390/ijms25073831

**Published:** 2024-03-29

**Authors:** Joanna Olejnik-Wojciechowska, Dominika Boboryko, Aleksandra Wiktoria Bratborska, Klaudia Rusińska, Piotr Ostrowski, Magdalena Baranowska, Andrzej Pawlik

**Affiliations:** 1 Department of Physiology, Pomeranian Medical University, al. Powstańców Wlkp. 72, 70-111 Szczecin, Poland; olejnikjoanna25@gmail.com (J.O.-W.); dominikaboboryko@gmail.com (D.B.); magdabaranowska29@gmail.com (M.B.); 2 Department of Internal Medicine, Poznań University of Medical Sciences, 60-356 Poznań, Poland; aleksandrabratborska@gmail.com; 3 Department of General Pathology, Pomeranian Medical University, al. Powstańców Wlkp. 72, 70-111 Szczecin, Poland; klaudia.rusinska@pum.edu.pl; 4 Department of Nursing, Pomeranian Medical University, Żołnierska 48, 71-210 Szczecin, Poland

**Keywords:** psoriasis, genetics, epigenetics, HLA, ncRNA, genome-wide association studies

## Abstract

Psoriasis is a chronic inflammatory skin disease, the prevalence of which is increasing. Genetic, genomic, and epigenetic changes play a significant role in the pathogenesis of psoriasis. This review summarizes the impact of epigenetics on the development of psoriasis and highlights challenges for the future. The development of epigenetics provides a basis for the search for genetic markers associated with the major histocompatibility complex. Genome-wide association studies have made it possible to link psoriasis to genes and therefore to epigenetics. The acquired knowledge may in the future serve as a solid foundation for developing newer, increasingly effective methods of treating psoriasis. In this narrative review, we discuss the role of epigenetic factors in the pathogenesis of psoriasis.

## 1. Introduction

Psoriasis is a chronic and recurrent inflammatory disease with an immune-mediated pathology. Its broad clinical spectrum can manifest from cutaneous psoriasis to psoriatic arthritis [1]. The prevalence of psoriasis worldwide is unevenly distributed, with a global prevalence rate of about 2–3% of the world’s population and higher incidence in Scandinavian countries, reaching as high as 11% [2,3].

The most common phenotype of psoriasis is plaque psoriasis, which is characterized by dry, silvery scales covering reddish patches of skin; other types include plaque, nail, guttate, inverse, and pustular psoriasis [4,5,6]. Extremely rare and the most severe type is generalized pustular psoriasis (GPP) [7].

Genetic factors strongly influence the occurrence of psoriasis; however, the fundamental genetic mechanisms are still unknown [8]. Research conducted on a population of twins emphasizes the significant role of environmental factors in the etiopathogenesis of psoriasis, as well as their interaction with genetic and immunological components [9,10]. Immunological aspects of psoriasis pathogenesis are associated with a major histocompatibility complex. Among the most highly linked psoriasis susceptibility alleles are HLA-Cw6, HLA-Cw1, HLA-Cw12, HLA-B27, and HLA-DR*07 [11,12,13,14,15].

Environmental factors, such as substance use, diet, stress, injuries, and infections, influence the development of psoriasis in genetically predisposed patients. These factors may trigger the disease in previously healthy individuals and contribute to psoriasis exacerbations [16].

The area of scientific research attempting to determine possible connections between genetics and the environment is epigenetics. Epigenetics explores the impact of molecular modifications on the regulation of gene expression [17]. When it comes to the pathogenesis of psoriasis, significant epigenetic changes include deoxyribonucleic acid (DNA) methylation, non-coding RNA regulation, and histone modification [18]. Moreover, several microRNAs (miRNAs) have been identified as being capable of regulating the balance between skin cells’ proliferation and differentiation, constituting the underlying cause of psoriatic lesions [19,20]. Due to the potential reversibility of epigenetic changes, its modification might serve as a promising target for the development of novel therapies.

**Figure 1:**
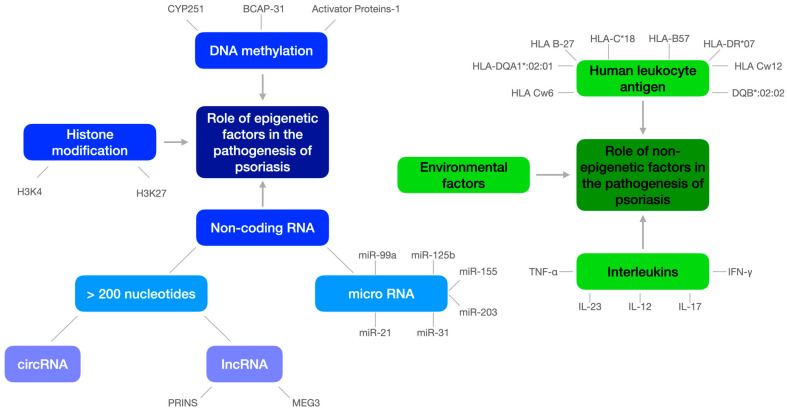
The role of epigenetic and non-epigenetic factors in the pathogenesis of psoriasis.

Figure 1 presents the role of epigenetic and non-epigenetic factors in the pathogenesis of psoriasis.

### The Significance of Antigen Presentation and Human Leukocyte Antigen (HLA) in Psoriasis

The immunological basis of psoriasis is the interaction of immune cells, mainly T lymphocytes and antigen-presenting cells, with epidermal keratinocytes. Several interleukins, such as IL-17, IL-23, IL-12, TNF-α, and INF-γ, mediate this interaction [21,22,23,24]. Abnormal leukocyte function and an inflammatory response targeting the patient’s epidermal cells results in keratinocyte hypertrophy and a shortened cell cycle. This process results in the formation of pathological skin lesions. Additionally, keratinocytes may generate cytokines and release autoantigens, further exacerbating the inflammatory cascade. Downregulation of pathways that inhibit inflammation shifts inflammation from acute to chronic [25].

The intensive development of genetics over recent years has made it possible to identify specific susceptibility alleles associated with the major histocompatibility complex (MHC) (Table 1). The best-known and most extensively described MHC-related allele in research is the HLA-Cw6 allele located on chromosome 6p21 (psoriasis susceptibility gene 1—PSORS1) [26]. The prevalence of this allele in the population ranges from 14.1% to 59.1% [27]. The understanding of its role in the pathomechanism of the disease remains limited. However, there are indications of its involvement in both innate and acquired immunity mechanisms. This involvement primarily manifests through its role in presenting antigens to T lymphocytes and its impact on NK cells, as documented in the literature [11]. The ability to interact via killer immunoglobulin-like receptors (KIRs) on NK cells may indicate a functional role for HLA-Cw6 in the immunopathogenesis of psoriasis [28]. HLA-Cw6 is a natural ligand for the inhibitory receptor KIR2DL1 and may also interact with receptors that activate NK cells. Dysfunction in NK cell regulation may be important in the pathogenesis of psoriasis [29]. Phenotypically, HLA-Cw6 is associated with droplet psoriasis. The HLA-Cw6 allele is frequently found in patients with obesity, exposure to stress, and a history of streptococcal pharyngitis. Skin lesions in individuals with this allele tend to be localized on the trunk, arms, and lower extremities [27,30]. Patients with the HLA-Cw6 variant respond better to treatment with methotrexate, biologic drugs directed against (IL)-12/23, IL-17, and IL-23 [12]. Given the heterogeneity of psoriasis pathogenesis and the differences in HLA-Cw6 positivity between ethnic groups, the role of HLA-Cw6 is more accurately assessed in clinical trials.

Another allele with a genetic link to the development of psoriasis is HLA-Cw1. Unlike HLA-Cw6, which is found in all ethnic groups, HLA-Cw1 is most common in the Asian population. Clinically, it is most commonly associated with erythroderma, pustular psoriasis, and psoriatic arthritis [24,25]. Family history is often positive and HLA-Cw1 patients respond less well to biologic therapy and show resistance to alefacept [31,32]. HLA-Cw1 also uses KIR receptors as ligands, in particular, KIR2DL2 and KIR2DL3 [33,34].

The frequency and dominance of HLA-C allele types very often varies between populations; in addition to the above, for example, HLA Cw12 is mainly found in the Turkish population [13]. Further HLA-Cs associated with the pathogenesis of psoriasis are still being sought.

HLA B-27 is a well-known marker of seronegative spondyloarthropathies and its association with psoriatic arthritis is widely reported in the scientific literature [35,36]. HLA-C*18 may also be associated with psoriasis. A strong association with the pathogenesis of psoriasis is also shown by HLA-B57, which is often associated with a severe clinical course of the disease [15]. HLA-DR*07 was found in patients with psoriatic arthritis and psoriasis involving the nails [37]. HLA-DQA1*:02:01 and DQB*:02:02 may be pharmacogenetic markers of response to azacitretin treatment [38].

**Table 1:** Human leukocyte antigen (HLA) alleles associated with psoriasis pathogenesis.

HLA Alleles	Type of Psoriasis	Ethnic Group Association	Drug Response
HLA Cw6	Droplet psoriasis Type I psoriasis [27]	In the general population varies from 14.1% to 59.1% [27]; more frequently in Caucasian patients than in the Asian population [27,39]	Responds better to treatment with methotrexate, biologic drugs directed against (IL)-12/23, IL-17, and IL-23 [12]
HLA Cw1	Erythroderma, pustular psoriasis, and psoriatic arthritis [12]	Most common in the Asian population [12]	Worse response to biologic therapy resistance to alefacept [31,32]
HLA Cw12	Severe psoriasis [13]	Most common in the Turkish population [13]	No research
HLA B-27	Psoriatic arthritis [35,36]	Diverse in ethnic group [35,36]	Predictor of good response to biological disease-modifying antirheumatic drugs (bDMARDs) [40]
Also associated with psoriasis pathogenesis: HLA-C*18, HLA-B57, HLA-DR*07, HLA-DQA1*:02:01 and DQB*:02:02 [15,37,38].			

## 2. Materials and Methods

An electronic literature search of the PubMed^®^ and Google Scholar^®^ databases was conducted. Search criteria included keywords “psoriasis”, “epigenetics”, “DNA methylation”, “non-coding RNA”, “microRNA”, “histones”, “enviromental factors”, and their combinations. Studies published up to January 2024 were considered. Inclusion criteria encompassed original genome-wide studies, reviews, systematic reviews, and meta-analyses specifically related to genetic or epigenetic studies of psoriasis. Duplicates and articles in a language other than English were excluded. For this review, an effort was made to select the most recent studies describing the issue. In the end, we selected 147 articles.

## 3. Future Outlooks in Psoriasis

The advancement of therapeutic approaches in psoriasis has undergone revolutionary progress in the past decade. The elucidation of the disease’s pathogenesis has been notably influenced by the development of epigenetics and advanced techniques for studying non-coding RNA, DNA methylation, and HLA.

A pivotal moment in human genome research was the implementation of genome-wide association studies (GWAS), where each gene was considered potentially associated with the disease [41]. By employing a meta-analysis of several million genetic markers and single-nucleotide polymorphisms, in conjunction with international datasets, researchers identified 65 genomic loci associated with psoriasis within the European population [42,43,44]. Individual alleles, coupled with extensive databases such as the UK Biobank [45], hold the potential to enhance the linkage to environmental risk factors, including smoking and stress [46].

A specific role is assigned to HLA-C*06:02, the identification of which has enabled the demonstration of its association with an earlier onset of the disease [47]. Further research is also recommended to evaluate the involvement of HLA alleles, considering the heterogeneous nature of psoriasis and different frequencies of occurrence in ethnic groups.

Uncovering new associations and effectively connecting them to other factors influencing the pathogenesis of the disease may facilitate accurate verification of the diagnosis. Identifying psoriatic skin lesions in the early stages of the disease can be challenging due to their resemblance to atopic eczema and seborrheic dermatitis. The phenome-wide association study (PheWAS) paradigm within electronic medical records (EMRs) has demonstrated the potential for a comprehensive catalogue of human diseases associated with published variants. The prevalence of disease alleles, in comparison to the odds ratio (OR), is still underestimated, and gaining a deeper understanding of psoriasis phenotypes should contribute to the improvement of disease classification and reduction in misdiagnoses [43,48]. Current forms of treatment, centered around biologic therapy, especially targeting IL-17 and IL-23p19, constitute a revolutionary approach. However, these therapies come with their limitations. Despite their significant impact, these drugs do not offer a complete cure and are associated with certain drawbacks such as potential side effects, suboptimal clinical responses in some individuals, and a decline in efficacy over time [49]. Consequently, the pursuit of better therapeutic targets with minimal side effects remains a desirable goal for the future.

Despite the discovery of potential mechanisms underlying psoriasis, numerous epigenetic patterns remain unexplained. The future appears to hinge on the utilization of genetic markers as potential prognostic factors to facilitate early detection and the initiation of treatment [50].

In a study by Natoli V. et al. [51], the investigation aimed to examine DNA methylation patterns in the cluster of differentiation of (CD)4+ T lymphocytes in patients with psoriasis and PsA. The results obtained may offer preliminary confirmation of the utility of DNA methylation patterns as potential biomarkers for distinguishing between skin psoriasis and PsA.

The current challenge involves integrating existing knowledge of genetic, genomic, and epigenetic changes along with environmental factors. This integration is crucial not only for treating psoriasis itself but also for addressing comorbidities. The prolonged elevation of proinflammatory cytokines and the chronic inflammation present in individuals with psoriasis contribute to an increased risk of developing metabolic syndrome (MS) and cardiovascular diseases, significantly impacting patients’ quality of life in the long term [52,53].

In psoriasis development, the role of microRNA remains invaluable. To date, numerous studies highlighting the crucial role of microRNA in the development of psoriatic lesions have been conducted. Further trials are needed to identify a possible novel therapy for psoriasis via regulation of microRNA expression.

The little-understood circular RNA (circRNA) also provides an avenue for further research. An expanding number of researchers suggest that dysregulation of circRNA is closely linked to severe diseases, including, but not limited to, autoimmune diseases. Previous studies demonstrate associated circRNA expression in psoriasis, regardless of the presence of skin lesions [54,55].

## 4. Epigenetic Changes in Psoriasis

### 4.1. DNA Methylation

DNA methylation is one of the earliest identified mechanisms of epigenetic modification in vertebrates. Its main form in mammals is 5-methylcytosine (5mC)—a compound formed in the catalyzed reaction by DNA methyltransferases (DNMT), transferring methyl groups (-CH3) to the cytosine ring in the presence of CpG dinucleotides. CpG density is a variability examined during DNA methylation studies. Regions with high CpG density in the genome are referred to as CpG islands [56,57]. DNA methylation directly influences the transcriptional level of genes—hypermethylation of CpG islands in gene promoter regions leads to repression, while hypomethylation leads to expression [58]. It also plays a role in maintaining chromosomal stability, regulating chromatin structure, and genomic imprinting [56]. Through these mechanisms, DNA methylation can be both a cause and a consequence of the progression of psoriasis.

It has been demonstrated that in psoriasis-affected skin, the gene expression of CYP2S1, ECE1, EIF2C2, MAN1C1, and DLGAP4 is negatively correlated with DNA methylation, with a particular focus on intragenic hypomethylation of CYP2S1, highlighting the need for further investigation in the future [58] (Table 2). CYP2S1 may regulate keratinocyte proliferation and also influence the immune response by inhibiting the expression of L1β, IL-8, IL-33, IL-36, LL37, CXCL10, and CCL20 genes [59].

In studies, demethylation of the BCAP31 promoter has also been confirmed, which is most likely the cause of increased expression of the BCAP31 protein in psoriatic keratinocytes and may play a role in the pathogenesis of their hyperproliferation and aberrant apoptosis [60]. The expression of B cell receptor-associated protein 31 (BCAP31) correlates with the characteristic phenomenon of parakeratosis in psoriasis [61].

DNA hypermethylation likely mediates the binding of proteins from the AP-1 family (FOS, FOSB, and JUND), influencing signaling pathways and playing a role in the formation of psoriatic Munro’s microabscess [62].

Particular attention also goes to the genes S100A9, SELENBP1, CARD14, KAZN, and PTPN22, whose loci overlap with the psoriasis susceptibility locus (PSORS), and their expression correlates with DNA methylation. In the study by Chandra et al., attention was drawn to the reversibility of DNA methylation, which may reflect periods of exacerbation and remission in psoriasis [63]. The reversibility of DNA methylation during psoriasis treatment and its potential association with the clinical picture were confirmed in a study using narrowband UVB phototherapy. It was demonstrated that the DNA methylation pattern had been reversed at the end of the entire phototherapy cycle, correlating with an improvement in the skin condition of the psoriasis-affected patients participating in the study [64].

### 4.2. Histone Modifications

Histones are proteins in the cell nucleus responsible for DNA packing, providing structural support for the chromosome, and regulating gene expression [18]. A nucleosome is a fundamental chromatin subunit consisting of a histone octamer of H2A, H2B, H3, and H4 proteins wrapped around 147 base pairs of DNA [65,66]. Post-translational modifications of histones, such as methylation, acetylation, phosphorylation, ubiquitylation, and ADP ribosylation, play a key role in many biological processes that affect DNA expression [67,68]. There are several histone modifications underlying the pathogenesis of psoriasis.

Histone methylation can lead to the activation or suppression of a state of transcriptional activity. The outcome of this modification depends both on the methylation site and the number of methyl groups introduced [69]. Typically, histone methylation occurs on the lysine and arginine side chains and several methyl groups can be introduced: mono-, di-, and tri-methylation are present [70]. The study has shown that a decrease in H3K9 dimethylation in psoriatic-altered keratinocytes correlates with increased IL-23 expression, which is sufficient to cause the psoriasis phenotype in a mouse model of psoriasis [71]. Increased H3K4 methylation, which may regulate PBMC gene expression, has been detected in the peripheral blood mononuclear cells (PBMCs) of psoriasis patients [72]. Additionally, the same study suggests that H3K4 and H3K27 methylation may affect patients’ response to biologic therapy in psoriasis [72].

Modification of H3K27me3 histone and enhancement of zeste homolog 2 (EZH2), a methylase of histone H3K27, is overexpressed in the psoriatic epidermis, whereas inhibition of EZH2 results in decreased histone H3K27me3 levels, reduced cell proliferation, and an alleviated phenotype in a mouse model of psoriasis [73]. Moreover, reduced levels of methylated H3K27, caused by overexpression of the demethylase Jmjd3, are associated with the differentiation of Th17 cells, which are involved in the pathogenesis of psoriasis [74]. 

Another histone modification that may be involved in the pathogenesis of psoriasis is acetylation. This process usually occurs on the lysine side chain of the N-terminal tail of histone proteins. The inclusion of an acetyl group, by weakening the histone–DNA bond, causes chromatin opening and facilitates transcription [68]. The enzymes responsible for histone acetylation are mainly histone acetyltransferases (HAT) and histone deacetylases (HDAC). These enzymes are dysregulated in psoriatic skin [75]. The study has shown that HDAC-1 is upregulated in lesioned skin compared to the healthy skin of control subjects, which may promote endothelial cell proliferation and keratinocyte survival [76]. The 2020 research [77] indicates that there is enhanced acetylation of H3K9 and H3K27 in the IL17A promoter region in the immune cells of psoriasis patients, which induced differentiation of Th17 and γδ T17 cells (which produce IL-17A γδ T cells), resulting in immune imbalance and disease progression. 

Additionally, decreased acetylation of histone H4 was observed in PBMCs of psoriasis patients, as well as a negative correlation between H4 modification and disease activity as measured by the psoriasis area and severity index (PASI) [72,78].

### 4.3. Non-Coding RNA

An growing body of clinical studies attests to the implication of non-coding RNA (ncRNA) in the pathogenesis of autoimmune diseases, notably psoriasis. Non-coding RNAs (NcRNAs) constitute a class of RNAs devoid of the capacity for protein translation. It is noteworthy that ncRNA constitutes over 98% of the human genome, whereas only approximately 2% of RNA is involved in encoding proteins [79]. Despite this limitation, they fulfill diverse regulatory roles in numerous biological processes by influencing gene expression and determining cellular protein localization and function. Categorized according to transcript size, ncRNAs are classified into two principal classes: long non-coding RNA (lncRNA) and microRNA (miRNA) [80,81].

#### 4.3.1. Long Non-Coding RNA

**Figure 2:**
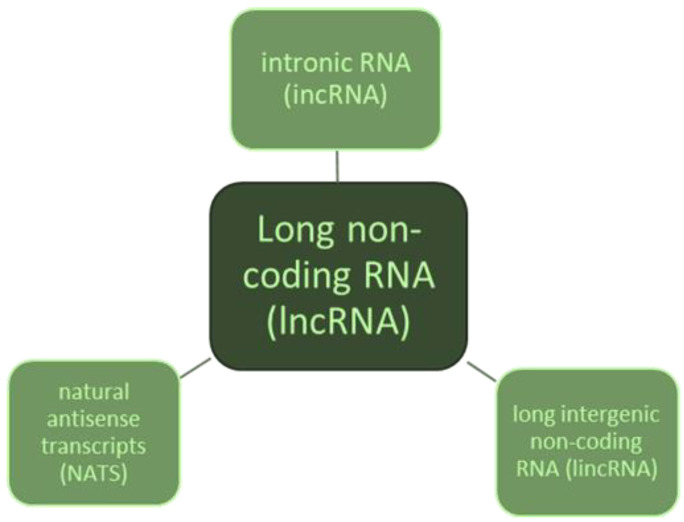
Classification of long non-coding RNA.

Long non-coding RNA (lncRNA) is a non-coding RNA segment formed in the transcription process, with a length exceeding 200 nucleotides. The number of human lncRNAs is estimated to be over 100,000, and detailed research on their significance and biological functions is still ongoing [82]. LncRNA can be divided into three groups: transcribed from gene introns—intronic RNA (incRNAs); transcribed from sequences complementary to protein-coding genes—natural antisense transcripts (NATS); and long intergenic non-coding RNA (lincRNAs) located between genes (Figure 2).

All these groups play significant roles, including regulation of cellular differentiation, apoptosis, and proliferation.

During molecular studies on the skin of psoriasis patients, a psoriasis-associated RNA induced by stress (PRINS) gene was identified, which is associated with susceptibility to psoriasis induced by stress, and it is recognized as a long non-coding RNA (lncRNA) [83,84]. In patients with psoriasis, the expression of PRINS is increased in both psoriatic keratinocytes and non-diseased skin cells. Additionally, PRINS expression decreases during effective psoriasis treatment. The study by Szegedi K et al. [85] demonstrated that the anti-apoptotic protein G1P6, regulated by PRINS, is induced in psoriatic keratinocytes, thereby inhibiting their spontaneous apoptosis. The level of G1P6 in hyperproliferative lesions is increased 400-fold compared to healthy epidermis. Another protein directly interacting with PRINS in the regulation of keratinocyte proliferation is nucleophosmin (NPM)—PRINS is a part of a regulatory complex formed with NPM. Immunohistochemistry revealed that the expression of NPM is elevated in the basal layer of psoriasis-affected skin samples, in contrast to samples from healthy, non-psoriatic skin [86].

Another lncRNA with a role in psoriasis pathophysiology is maternally expressed gene 3 (MEG3). MEG3, by regulating miR-21 and affecting caspase-8 expression, can inhibit psoriatic keratinocyte proliferation and promote apoptosis. However, MEG3 expression in psoriatic skin is significantly reduced [87,88].

Qiao M et al. developed an in vitro model in which, upon IL-22 stimulation of keratinocytes, they discovered that long non-coding RNA pseudogene 1 homeobox 2 Msh (MSX2P1) facilitated the progression and growth of keratinocytes. MSX2P1 served as an endogenous sponge directly binding to miR-6731-5p and activating S100A7. This leaves room for speculation that the biological network involving MSX2P1-miR-6731-5p-S100A7 could potentially serve as a novel therapeutic target in future psoriasis treatments [89].

#### 4.3.2. Circular RNA

Circular RNA (circRNA), similar to lncRNA, is characterized by length exceeding 200 nucleotides. However, unlike linear transcripts, circRNA possesses a circular structure, attributed to the covalent linkage of its 3′ and 5′ ends. This circular conformation provides circRNA with greater resistance to endonucleases compared to linear transcripts. The inherent stability of circRNA has prompted efforts in modern medicine to explore its potential use as a biomarker [90].

CircRNAs can regulate host gene expression through RNA modification, as evidenced by a study conducted by Yang L et al. [91]. The study revealed the enrichment of hsa_circ_0004287 in peripheral blood mononuclear cells (PBMCs) from individuals with psoriasis. Hsa_circ_0004287 was found to inhibit skin inflammation by interacting with MALAT1 in an m6A-dependent manner. This discovery holds potential for future consideration as a focal point in the treatment of psoriasis.

Additionally, there is potential for circular RNA 0061012 (circ_0061012), ciRS-7, and circZRANB1 to serve as biomarkers for psoriasis in the future [54,92].

#### 4.3.3. The Essential Role of microRNAs in Psoriasis

Recent research has increasingly focused on the role of microRNAs (miRNAs) in the development and progression of inflammatory dermatological conditions, including psoriasis. Although miRNAs are very short and consist of approximately 22 nucleotides, they play an essential role in the post-transcriptional regulation of gene expression [93]. For this reason, they have a profound impact on the immune system and inflammatory responses in the skin. To date, studies have identified numerous miRNAs that are dysregulated in psoriasis and have been implicated in various processes involved in psoriasis pathogenesis, including aberrant keratinocyte proliferation, dysregulated immune responses, and altered cytokine production.

To begin with, miR-21 is among the most studied miRNAs and has been proven to be increased in psoriasis [94,95]. It suppresses apoptosis in activated T cells and contributes to their prolonged survival and, therefore, to persistent inflammation [95]. Furthermore, miR-21-3p, a novel miRNA induced by UV exposure, promotes inflammation in keratinocytes and exacerbates psoriatic lesions [96]. The miR-21 overexpression correlates with excessive proliferation, disturbed differentiation, and inhibition of apoptosis of skin cells [88]. In addition, miR-31 is also upregulated in psoriatic patients and promotes epidermal proliferation [20]. Because it also enhances the inflammatory reactions in the skin and augments leukocyte chemotaxis, it is advisable to target its expression as a novel therapy to alleviate psoriasis severity. Research has shown that miR-31 is responsible for regulating the expression of inflammatory mediators by affecting the activity of the serine/threonine kinase 40 (STK40) gene in keratinocytes. Therefore, overexpression of miR-31 promotes inflammation in the affected skin [97]. In addition, inflammatory cytokines activate nuclear factor kappa-light-chain-enhancer of activated B cells (NF-κB), which induces miR-31 transcription and, through this induction, also inhibits protein phosphatase 6 catalytic subunit (PPP6C) expression. Lower expression of this gene consequently contributes to epidermal hyperproliferation in psoriasis [20]. Moreover, significant negative correlation was found between miR-31 and endothelin-1 (ET-1), which through being produced by psoriatic keratinocytes causes suppression of their apoptosis [98].

Furthermore, in psoriatic patients, miR-155 levels are elevated in peripheral mononuclear cells, and it shows a positive correlation with the disease severity. It upregulates TNF-α expression and increases lymphocyte infiltration [94]. Therefore, it might constitute a relevant marker of skin inflammation in psoriasis.

Increased expression of miR-203 has been observed in pathologically altered keratinocytes, which, by directly affecting multiple epidermal genes, influences the proliferation and differentiation of these cells, therefore being involved in the pathogenesis of psoriasis [99,100]. miR-203 is the first identified skin-specific miRNA that regulates keratinocyte differentiation in a protein kinase C-dependent manner [100]. The recent study has shown that miR-203 significantly downregulates the expression of liver X-α receptor (LXR-α) and peroxisome proliferator-activated receptor-γ (PPAR-γ) in psoriatic lesions, leading to a hyperproliferative keratinocyte phenotype in this skin disease [101]. Moreover, the p63 protein, as a member of the p53 protein family, is one of the most significant regulators of epidermal morphogenesis and homeostasis [102]. miR-203, by binding to the 3’ UTR of p63 mRNA, regulates the level of p63 expression and affects the p63-dependent proliferative potential of epithelial precursor cells in its development and keratinocyte differentiation [102,103].

Another microRNA involved in psoriasis pathogenesis is miR-99a, which is downregulated in psoriatic dermatic lesions [104,105]. miR-99a inhibits HaCaT cell proliferation by suppressing the expression of FZD5 and FZD8 through direct binding to the 3’UTR of target genes, as well as by reducing protein levels of β-catenin and cyclin D1 [106,107]. Furthermore, miR-99a inhibits the expression of insulin-like growth factor 1 receptor (IGFR-1), which is also involved in skin development and the pathogenesis of psoriasis [104].

Another downregulated miRNA in psoriasis is miR-125b. This type of microRNA is lower in serum and skin samples of patients suffering from psoriasis compared to control groups. This report also revealed that there is a statistically significant negative correlation between miR-125b and PASI [19]. Inhibition of miR-125b results in upregulation of fibroblast growth factor receptor 2 (FGFR2), leading to hyperproliferation of keratinocytes, but not directly to changes in their differentiation [108]. Another target gene for miR-125b is AKT3. Overexpression of miR-125b by blocking the AKT pathway inhibits the proliferation of human epidermal keratinocytes (HEKs) [109]. Furthermore, miR-125b targets ubiquitin-specific peptidase 2 (USP2), the removal of which enhances the differentiation of keratinocytes [110]. Summarizing all the aforementioned effects of miR-125b, it can be concluded that higher expression of this miRNA may be an important factor in the prevention of psoriasis [107].

In summary, upregulated miR-31/miR-203/miR-155/miR-21 and downregulated miR-99a/miR-125b are epigenetic factors that contribute to processes underlying the pathogenesis of psoriasis, such as aberrant differentiation and excessive proliferation of skin cells [107] (Table 3).

## 5. Environmental Factors

Although psoriasis was historically considered to be mainly hereditary, the current understanding recognizes the pivotal role that environmental factors play in determining its natural history [111]. Environmental risk factors are now recognized as dynamic elements, either exacerbating disease in diagnosed individuals or catalyzing disease development in genetically susceptible individuals [112,113].

### 5.1. Bacterial Infections

Bacterial infections have been considered a potential risk factor for developing psoriasis, with certain pathogens like Streptococcus and Staphylococcus being connected [114,115,116,117]. These infections can initiate or intensify the condition by initiating inflammatory responses and altering the skin microbiome [118,119,120]. Aly R. and colleagues were the first to report that Staphylococcus aureus counts were higher on psoriatic plaques in comparison to normal skin. Moreover, the total bacterial counts were raised on plaques. Furthermore, 80% of the S. aureus strains taken from psoriatic patients were found to be resistant to 10 units of penicillin [118]. Based on their findings, the study concludes that psoriatic skin represents a public health hazard due to increased desquamation.

Rademaker M. et al. previously demonstrated differences in skin microbiomes between psoriasis patients and non-psoriasis patients. However, the significance of this difference remains unclear [121].

Additionally, there is an independent but slight correlation between psoriasis and an increased risk of severe infection. Patients with psoriasis who are taking immune-modulatory medication should typically avoid getting live vaccinations, such as MMR, varicella, zoster, and yellow fever. However, this depends on the level of immune suppression and individual risk factors [121].

In addition, disruption of the skin and gut microbiomes and systemic inflammation are associated with psoriasis, which may facilitate the translocation of bacteria into the bloodstream. The pathophysiology of psoriasis may involve dysbiosis of the skin microbiota and gut microbiota; hence, modifying the flora in the intestines and employing suitable antibiotics may be useful treatment approaches. The presence of latent or nonreplicating bacteria in the systemic circulation, as well as iron dysregulation, may have implications for the development and management of psoriasis [117].

However, the available evidence lacks consistency, highlighting the need for further research to fully comprehend the role of bacterial infections in psoriasis development and progression.

### 5.2. Obesity and Diet

As an increasingly common modern ailment in developed nations, obesity presents a multitude of negative outcomes and health issues. Individuals with psoriasis have a higher likelihood of developing obesity and other metabolic disorders [122]. The condition is both more prevalent and severe among those who are obese, in addition to impacting their response to treatment.

The association between obesity and psoriasis is thought to be due to chronic low-grade inflammation, which is a common pathophysiological mechanism in both conditions [123,124]. Weight reduction has been shown to improve the severity of psoriasis in overweight individuals [122,125]. The role of diet in psoriasis is less clear, but it is suggested that dietary habits and lifestyle may contribute to the progression of both obesity and psoriasis [124].

Research consistently shows that a poor diet, particularly one high in saturated fatty acids, simple sugars, and red meat, can exacerbate psoriasis [126]. Conversely, diets rich in n-3 polyunsaturated fatty acids, antioxidants, and anti-inflammatory nutrients, such as those found in fish, fruits, and vegetables, may help alleviate symptoms [127].

Obesity, often a result of a poor diet, is a significant risk factor for psoriasis, and weight loss interventions have been shown to improve the condition [125].

### 5.3. Traumatic Skin Injuries

Research suggests that traumatic injury to the skin may be a risk factor for the development of psoriasis, particularly psoriatic arthritis [128,129,130].

There are at least two theories explaining how trauma could contribute to psoriatic arthritis formation. These theories center on the “deep Koebner effect”, the idea of a synovioentheseal complex [131], and the biomechanical activation of the innate immune system with neuropeptides.

The “deep Koebner” effect may occur in psoriatic arthritis (PsA), similar to the Koebner phenomenon observed in psoriasis. The Koebner phenomenon refers to the development of psoriatic lesions at sites of injury or trauma. In the case of PsA, it is theorized that traumatic injury may trigger an inflammatory response in genetically susceptible individuals, leading to the activation of the innate immune system. This immune response may then interact with the adaptive immune system, particularly T cells, which can produce cytokines and further contribute to the development of PsA. The exact mechanisms of how the deep Koebner effect triggers PsA are still not well understood.

In the case of activation of the innate immune system by biomechanical factors, nerve endings may release neuropeptides in response to trauma [132,133]. The upregulation of neuropeptides, such as substance P and vasoactive intestinal peptide, which can be found in both PsA-affected synovium and psoriatic skin lesions, supports this theory [134,135]. Substance P, once released, stimulates the proliferation of synoviocytes, subsequently resulting in the release of PGE2 and collagenase, which causes the inflammatory cascade [136,137].

However, the exact mechanism by which trauma may trigger psoriasis is not fully understood, and further research is needed to explore this relationship.

### 5.4. Alcohol Consumption

Alcohol consumption has a range of toxic effects on the human body, including interference with hepatic metabolism and immunological functions. Recent scientific research suggests that consuming an excessive amount of ethanol may also increase the likelihood of developing skin conditions, such as rosacea, urticaria, and psoriasis, particularly in men [138,139,140].

Chronic alcohol consumption causes an overabundance of keratinocyte growth and increases the levels of tumor necrosis factor-α (TNF-α) and T lymphocyte activation. These components influence immune system functions and elevate the likelihood of acquiring psoriasis. This association is further supported by the higher prevalence of psoriasis in patients with alcoholic liver disease [141]. The risk appears to be particularly high with nonlight beer intake [138].

Additionally, if alcohol consumption is continued during treatment, traditional medications used in psoriasis treatment (such as retinoids, methotrexate, and cyclosporine) may exhibit lower efficacy or result in severe side effects such as liver fibrosis [142].

## 6. Conclusions

The role of epigenetic modifications in the pathogenesis of psoriasis is significant, as evidenced by the understanding of the mechanisms discovered so far [143]. Further research is necessary to determine whether a specific epigenetic change plays a role in the development of the disease or is a consequence of it. A suitable direction for future research may also involve monitoring the interactions of epigenetic factors, including expanding knowledge about the interaction of circRNA as miRNA sponges. The acquired knowledge may serve as a hint for continuously evolving diagnostic methods and treatment approaches [144,145]. This knowledge may also prove to be a factor in helping to assess prognostic factors in the diagnosis of psoriasis [146,147]. Studies focused on assessing cardiovascular events and other cardiovascular diseases in patients with psoriasis deserve special attention; epigenetic factors, still being discovered, may become biomarkers of cardiovascular risk in patients with psoriasis in the future.

Understanding the mechanisms of epigenetic modifications in psoriasis also emphasizes the role of lifestyle medicine. Proper education of patients about a balanced diet, maintaining a healthy body weight, avoiding substances, or even proper care of the affected skin is important to prevent secondary bacterial infections. Taking care of these factors can prevent the cascade of epigenetic factors leading to keratinocyte hyperproliferation, parakeratosis, or inhibition of cell apoptosis. Caring for elements of lifestyle medicine in patients with psoriasis also offers the opportunity for a better response to treatment.

## Figures and Tables

**Table 2:** Gene expression correlated with DNA methylation in psoriasis.

Gene Regulation in Psoriasis	Hypomethylation	Hypermethylation
CYP2S1	+	
ECE1	+	
EIF2C2	+	
MAN1C1	+	
DLGAP4	+	
BCAP31	+	
AP-1 family (FOS, FOSB, and JUND)		+
S100A9	+	
SELENBP1		+
CARD14		+
KAZN		+
PTPN22	+	

**Table 3:** MicroRNA involved in psoriasis pathogenesis.

microRNA	Level	Potential Effect
miR-21	⬆	Suppression of activated T cells’ apoptosis, enhanced keratinocyte proliferation
miR-31	⬆	Keratinocyte proliferation, leukocyte chemotaxis stimulation, promoting inflammation and proliferation of keratinocytes
miR-155	⬆	TNF-α expression upregulation, stimulation of lymphocyte infiltration
miR-203	⬆	Regulation of keratinocyte proliferation and differentiation
miR-99a	⬇	Inhibition of HaCaT cell proliferation, downregulation of IGFR-1 expression
miR-125b	⬇	Keratinocyte hyperproliferation

## Data Availability

Not applicable.

## Abbreviations

GPP	generalized pustular psoriasis
miRNA	mikroRNA
lncRNA	long non-coding RNA
lincRNA	long intergenic non-coding RNA
circRNA	circular RNA
MHC	major histocompatibility complex
PSORS1	psoriasis susceptibility gene 1
KIR	killer immunoglobulin-like receptor
bDMARD	biological disease-modifying antirheumatic drugs
DNMT	DNA methyltransferases
BCAP31	B cell receptor-associated protein 31
PSORS	psoriasis susceptibility locus
PBMCs	peripheral blood mononuclear cells
EZH2	enhancer of zeste homolog 2
HAT	histone acetyltransferases
HDAC	histone deacetylases
PASI	psoriasis area severity index
NATS	natural antisense transcripts
PRINS	psoriasis-associated non-protein coding RNA induced by stress
NPM	nucleophosmin
MEG3	maternally expressed gene 3
MSX2P1	pseudogene 1 homeobox 2 Msh
MALAT1	metastasis-associated lung adenocarcinoma transcript 1
STK40	serine/threonine kinase 40
ET1	endothelin 1
PPAR-γ	peroxisome proliferator-activated receptor-γ
LXR-α	liver X-α receptor
IGFR-1	insulin-like growth factor 1 receptor
FGFR2	fibroblast growth factor receptor 2
HEK	human epidermal keratinocytes
USP2	ubiquitin-specific peptidase 2
GWAS	genome-wide association studies
PheWAS	the phenome-wide association study
EMR	electronic medical records
OR	odds ratio
MS	metabolic syndrome
